# Making sense of how HIV kills infected CD4 T cells: implications for HIV cure

**DOI:** 10.1186/2052-8426-2-20

**Published:** 2014-07-03

**Authors:** Nathan W Cummins, Andrew D Badley

**Affiliations:** Division of Infectious Diseases, Mayo Clinic, 200 - 1st Street SW, Rochester, MN 55905 USA

**Keywords:** HIV, Cure, Apoptosis, Pyroptosis, Caspase 1, DNA-PK, Casp8p41, IFI16, Inflammation

## Abstract

Defining how HIV does, and does not, kill the host CD4 T cell that it infects is of paramount importance in an era when research is approaching a cure for infection. Three mutually exclusive pathways can lead to the death of HIV-infected cells during the HIV life cycle, before, coincident and after HIV integration and consequently may affect viral replication. We discuss the molecular mechanism underlying these pathways, the evidence supporting their roles *in vivo*, and contemplate how understanding these pathways might inform novel approaches to promote viral cure of HIV.

## Review

### Introduction

In the past 33 years of the HIV pandemic, there has been monumental progress in the clinical management of HIV disease. Once considered a death sentence due to the inexorable decline in CD4 T cell number and function over time ultimately leading to AIDS, patients with HIV infection who have access to effective antiretroviral therapy (ART) now have a near normal life expectancy [1, 2]. In the first 15–20 years of the pandemic, during a time when effective ART was unavailable or in its infancy, there was intense scientific interest and inquiry into molecular mechanisms of HIV replication, in order to develop effective drugs with which to inhibit HIV replication *in vivo*. In the past 15 years, multiple drugs in multiple classes have been developed and much has been learned about how best to use combinations of agents. Now it is possible, and even expected, that clinical viral suppression can be achieved, with consequent immune reconstitution. However treated individuals still have excess morbidity and mortality when compared to uninfected persons, due largely to accelerated aging and age related diseases such as cardiovascular disease [3], metabolic syndrome [4], solid organ malignancies [5], neurocognitive and functional decline [6] and osteoporosis [7, 8].

**Figure 1:**
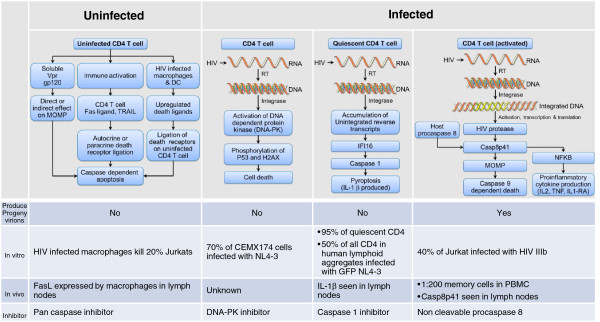
**Pathways of CD4 T cell death in HIV infection.** Depicted are the described pathways for both uninfected and infected CD4 T cell death in the context of HIV infection and whether progeny virions are produced as a result of the pathway. Examples are provided describing the pathogenicity of the pathway in relevant *in vitro* models. Specific *in vivo* support of the pathwaysm biologic relevance are also described. Theoretical ways to inhibit the described pathways are conjectured. References [26–28, 37–44].

In the past two years, there has been a fundamental shift in the focus of HIV research, now aimed directly at achieving either a sterilizing cure (eradication of HIV from cells and tissues) or a functional cure (spontaneous control of HIV such that ART is not needed to preserve immune function). Long considered quixotic, the search for a cure for HIV has been spurred by the case reports of a small number of patients who have had HIV eradicated [9, 10]. The central enigma of strategies attempting to achieve a sterilizing cure is that while HIV virions and many HIV proteins individually can cause death of immune cell subsets, a subset of infected cells do not die. Yet, in order to cure HIV, all (or nearly all) HIV infected cells must die, while preserving as many non-infected cells as possible. This has proven to be a difficult task, and has brought back into focus research on how HIV-infected cells die, and how that might be manipulated to achieve a cure. It is therefore of great interest to understand how cells which contain HIV die (Figure 1).

### Both HIV-infected and uninfected CD4 T cells die during HIV infection

HIV induces cell death in both uninfected and infected T cells. The mechanisms of uninfected T cell death during HIV infection have been reviewed extensively elsewhere [11]. Briefly, so-called "bystander" T cells die from aberrant apoptosis induced by a number of stimuli, including over-expression of death ligands (Fas Ligand, TNF and TRAIL) on immune cells; direct cytotoxicity of a number of soluble HIV proteins (e.g. Gp120, Tat, Nef, Vpr); and activation-induced cell death driven by a chronically activated and hyper-inflammatory immune state associated with HIV infection. It is quite possible that these inducers of cell death also affect HIV-infected cells, but by their nature are not unique to HIV-infected cells. Other cell death mechanisms, for instance autophagy, have been described in the context of *in vitro* HIV infection, but have not yet been defined with regard to infected or bystander cell death [12, 13]. A number of studies have observed that uninfected T cell death predominates in untreated HIV infection, and that the death of these uninfected cells drives the immunodeficiency associated with untreated HIV disease [14–16]. Consistent with this model, it is clear that aberrant apoptosis, including that of uninfected cells, is significantly reduced with virologic suppression by antiretroviral therapy [17, 18], which is a *sine qua non* of investigative cure strategies. However, in order to achieve a cure for HIV infection, strategies must be developed which favor the death of those cells which despite treatment, still contain HIV.

One may question what evolutionary advantage is gained by a retrovirus inducing apoptosis in a cell that it infects. In fact, the significant presence of endogenous retroviruses in the human genome [19] and the presence of natural, non-progressive SIV infections in non-human primates [20] argue in favor of evolutionary co-adaptation to avoid infected cell apoptosis. However, our group and others have shown that HIV-induced cell death actually increases HIV replication via NF-κB activation by the Bcl10/MALT1/CARMA complex, a process which is dependent on active caspase 8 [21–24]. This suggests that the virus has evolved in a way to overcome the potential replication-limiting outcome of infected cell death.

### A tripartite approach to infected cell death

Infection of a CD4 T cell by HIV results in one of three outcomes. If the cell is not permissive to infection, either due to activation state or expression of pre-integration cellular restriction factors, the cell is abortively infected, i.e. the viral life cycle ends before integration into the host genome. If the cell is permissive to infection, following attachment, the viral life cycle is completed and progeny virions are produced, and that cell is considered productively infected; in some cases productively infected cells can revert to latency wherein viral proteins are no longer produced. In a permissive cell, when HIV infection and integration occurs, in some circumstances no proviral transcription occurs, no viral proteins are transcribed, and the cell is considered latently infected. The existence of these long-lived latently infected CD4 T cells represents the major obstacle to HIV eradication, as they have long half-lives, do not replicate HIV and therefore are not impacted by HIV therapies which target viral proteins, and are not targeted by host immune mechanisms which target HIV antigens. Moreover the potential presence of productively infected cells which could continuously replenish the latent reservoir may present further challenges for HIV eradication strategies; although mathematical models suggest that these productively infected cells may not contribute significantly to the latent reservoir [25]. Finally, a number of endogenous and exogenous factors can reactivate viral transcription in latently infected cells, which re-establishes productive infection.

### Pre- integration HIV-induced infected cell death

A series of studies [26–28] have convincingly described how CD4 T cells abortively infected with HIV die secondary to caspase-1 dependent pyroptosis, a form of inflammatory programmed cell death. Using human lymphoid aggregate cultures (HLAC) infected with a GFP-expressing HIV, the majority of CD4 T cells that die in the *ex vivo* system do not have integrated HIV nor do they express GFP, and hence are not productively HIV-infected [26]. Cell death does require HIV entry, as inhibitors of HIV entry, including AMD3100 (co-receptor CXCR4 inhibitor) and T20 (viral gp41 inhibitor) prevent CD4 T cell depletion in the infected HLACs. Interestingly, non-nucleoside reverse transcriptase inhibitors (NNRTIs) and integrase inhibitors (INSTIs) also inhibit CD4 T cell depletion, but not nucleoside RTIs (NRTIs), suggesting that accumulation of early reverse transcripts is toxic in abortively infected cells. Dying cells but not the surviving cells have activation of caspase-1 and caspase-3, as indicated by FLICA™ probes and enzymatic activity assays, and consequently produce interleukin-1β as a consequence of caspase-1 activation. Activation of both caspase-1 and caspase-3 define pyroptosis, (as opposed to apoptosis) and it is a prototypic, but not the only, form of cell death that is associated with inflammation.

Using DNA affinity chromatography and mass spectrometry, interferon-γ-inducible protein 16 (IFI16), a component of the inflammasome [28], was identified to bind HIV-1 Nef DNA. shRNA against IFI16 abrogates activation of caspase-1 after HIV infection and protects CD4 T cells from death in the HLAC model. When added to the HLAC model, caspase-1 inhibitors inhibit cell death in abortively infected, resting CD4 T cells [27], whereas inhibitors of caspase-3 and caspase-6 as well as necrostatin (a RIPK1 inhibitor) do not. These results were confirmed using shRNA-mediated knockdown. Notably, a minority of cell death in the HLAC model occurred in activated productively infected CD4 T cells, which was blocked by selective caspase-3 inhibitors.

A number of host cellular proteins have been demonstrated to restrict HIV replication events prior to HIV integration. APOBEC3G is a cytidine deaminase that induces G to A hypermutation in viral DNA. This effect can be counteracted by HIV-1 protein Vif (virion infectivity factor) [29–31]. TRIM5alpha is another cellular innate antiviral protein, which disrupts the uncoating process of HIV-1 capsid weakly inhibiting successful reverse transcription [32]. P21, a cyclin dependent kinase inhibitor, likely acts as a restriction factor by several reported mechanisms, including blocking HIV integration in primitive hematopoietic cells [33, 34]; inhibiting dNTP biosynthesis by repressing ribonucleotide reductase 2 [35]; and regulating phosphorylation of SAMHD1 [36]. Together, these pre-integration restriction factors potentially serve to prevent HIV-infected cell death.

### HIV-integration induced infected cell death

An alternate mechanism of HIV-infected cell death is dependent upon the host cell response to HIV integration [37]. Using *in vitro* infection of T cell lines and activated primary CD4 T cells, in contrast to the findings described above, all three classes of ARV: NRTIs, NNRTIs and INSTIs, (plus a protease inhibitor [PI] to prevent spreading infection), inhibited HIV-induced cell death. Furthermore, wild type, Tat-deficient and Rev-deficient HIV all caused HIV-infected cell death whereas integrase-deficient HIV did not, suggesting that integration was necessary for HIV-induced cell death in activated cells. Accordingly, pharmacologic inhibition of integrase activity with INSTIs in *ex vivo* HIV-infected primary cells decreased cell death. Wild type, but not integrase-deficient HIV, caused phosphorylation and activation of DNA-dependent protein kinase (DNA-PK), a cellular censor of double stranded DNA breakage. DNA-PK activation leads to phosphorylation and activation of p53 and initiation of p53-dependent cell death pathways. Pharmacologic inhibition of DNA-PK decreased both activation of p53 and CD4 T cell death, solidly implicating this pathway in HIV-infected cell death in activated cells.

### Post-integration HIV-induced infected cell death

A final mechanism of cell death occurs exclusively in productively HIV-infected cells through a process which requires HIV protease. It has long been observed that expression of HIV protease in cells is an intrinsically cytotoxic process [38]. A series of studies using cell free lysates, knockout cells, and *in vitro* digestion followed by mass spectroscopy, demonstrate that HIV protease, which is known to have a degenerate substrate specificity, cleaves the cellular protein procaspase-8 between amino acids 355 and 356 to generate a novel caspase-8 fragment, termed Casp8p41 [39–41]. Casp8p41 is missing the catalytic cysteine at position 360, and therefore does not have intrinsic proteolytic activity. However expression of Casp8p41 independently induces activation of NF-κB, which in turn drives both expression of pro-inflammatory cytokines [42] and up-regulates HIV-LTR transcription [23]. In addition, Casp8p41 translocates to the mitochondria, where it induces depolarization of the mitochondrial outer membrane, resulting in release of cytochrome c and activation of caspase-3 and initiates the phenotypic changes of apoptosis [43], in a Bax/Bak and pro-caspase 9 dependent manner [44]. T cell lines genetically deficient in procaspase-8 are partially resistant to HIV-induced cell death, while reconstitution of these cells with wild type procaspase-8 restores HIV-induced cell death; whereas cells reconstituted with procaspase-8 that cannot be cleaved by HIV protease do not fully recapitulate the wild type phenotype [41].

### Relevance of HIV-infected cell killing *in vivo*

An abundant and inconvenient truth concerning apoptosis is that it is extremely difficult to determine how a cell has died after death has begun, and the cellular features of death are underway. This is because most forms of cell death (including forms that occur as a consequence of HIV infection) cause activation of nucleases and proteases, which allow detection of the dying cell (through techniques such as TUNEL staining, or identifying the presence of active Caspase 3). However these same nucleases and proteases inhibit detection of HIV nucleic acids or HIV proteins, which are necessary to define which cells are and are not infected with HIV. Thus direct demonstration that a cell is HIV-infected (i.e. contains HIV DNA or RNA or protein), is dying (is TUNEL positive), and has died due to a particular pathway (such as Fas ligation) is technically difficult, if not impossible. This is true both *in vitro* and especially *in vivo*.

To search for the relevance of the three pathways of infected cell death described above, most studies involve interrogation of specific markers within lymphatic tissues or blood from HIV-infected persons. Active caspase-1 and IL-1β have been observed by immunohistochemistry (IHC) of a lymph node from a viremic HIV-infected patient [27]. However, it is unknown whether the caspase-1 and/or IL-1β positive cells were abortively infected or if another stimulus, such as TLR4 stimulation from lipopolysaccharide could have induced inflammasome activity. Similarly, while active p53 has been demonstrated in peripheral blood mononuclear cells and lymph nodes from HIV infected patients [45, 46], since p53 can be activated by a numerous stimuli, it is unknown whether its activation in these patients was due to HIV integration and DNA-PK. Conversely, since Casp8p41 is only generated by HIV protease, its detection is by definition evidence of active HIV protease, which only occurs in HIV-infected cells. Casp8p41 is detectable by neoantigen specific monoclonal antibody IHC in lymph nodes from HIV-infected subjects, where Casp8p41 expression co-localizes with active caspase-3 [41]. Casp8p41 is also detectable in PBMCs from HIV-infected subjects, particularly in the memory subset of CD4 T cells [47]. In viremic patients, Casp8p41 expression in memory CD4 T cells is inversely associated with CD4 T cell count, and decreases in Casp8p41 expression after initiation of ART are associated with increases in CD4 T cell count [47]. In addition, persistent expression of Casp8p41 in memory CD4 T cells in virologically suppressed patients is associated with increased risk of CD4 T cell declines over time, despite continued virologic suppression [48]. Finally, mutations in HIV protease that are over-represented in patients with preserved CD4 T cell counts despite virologic failure of ART, when introduced into HIV protease, have an impaired ability to cleave procaspase-8 and generate Casp8p41 despite cleaving Gag/Pol normally [49], suggesting that impaired Casp8p41 production contributes to preserved CD4 T cell counts in some patients with resistance to ART.

### Relevance of Infected cell killing to HIV pathogenesis and the HIV cure agenda

Since the IFI16 and DNA-PK death pathways are dependent on viral attachment and entry, yet kill cells prior to or coincident with integration and HIV replication, they likely contribute to CD4 depletion and immunodeficiency that occur during untreated HIV infection when robust viral replication occurs. In the setting of suppressive antiretroviral therapy, when viral replication is decreased by orders of magnitude, new rounds of viral attachment and entry are rare events and therefore cell death by IFI16 and DNA-PK are also likely rare events. By definition, the cells which are latently HIV infected and contain integrated HIV DNA have already survived through the points in the viral life cycle when IFI16 and DNA-PK mediated killing occur. By contrast, the Casp8p41 death pathway is always a rare event – typically less that 0.1% of memory CD4 T cells [47]. Unlike pretranscriptional pathways of HIV infected cell death however, the Casp8p41 pathway is likely of relevance, or potential relevance, in latently-infected cells induced to reactivate virus. Indeed, since HIV reactivation does not result in the death of reactivating cells [50, 51], yet it does produce progeny virions (*de facto* demonstrating that HIV protease is active), it is of pressing interest to understand whether these cells are capable of generating Casp8p41, and why these cells do not die in a Casp8p41 (or any other pathway) -dependent manner.

### Potential to manipulate HIV infected cell death pathways

Understanding these pathways by which HIV causes infected cells to die, allows studies designed to modify these pathways, for the potential therapy of HIV. Small molecular inhibitors of procaspase-1, including VX-740, VX-765 and IDN-6556, are in various stages of clinical development for a number of inflammatory disorders [52–54]. Small molecular inhibitors of DNA-PK are in early development but have yet to enter clinical trials due to poor pharmacokinetic parameters of current agents [55]. Theoretically these two classes of agents may be able to inhibit pyroptosis and apoptosis of cells induced to die by IFI16 and/or DNA-PK dependent pathways respectively. Cleavage of procaspase-8 by HIV protease is inhibited by HIV-protease inhibitors (PIs) [56], for which there is ample data demonstrating improvements in CD4 T cell number [57], including improvements in CD4 T cell numbers independent of antiviral effect [58]. However there are numerous off-target effects of PI, including intrinsic anti-apoptotic effects, which likely confound these observations. Assuming that Casp8p41 could be selectively inhibited, inhibiting it or any of the HIV-infected cell death pathways would predictably lead to increased CD4 T cell counts, yet these cells would still be infected, and in our opinion would likely be inappropriately activated and likely anergic, and dysfunctional. From a virologic standpoint, inhibiting cell death induced by any of these pathways might also be counterproductive. Inhibiting death of infected cells, either pre or post integration would predictably increase the pool of cells which contain cell associated HIV DNA, thereby hindering attempts at eliminating that reservoir.

Favoring death of infected cells, by enhancing infected cells sensitivity to apoptotic stimuli, though, may be more attractive. This hypothetical model by which to eradicate HIV has been previously proposed as the "Prime, Shock and Kill" model [59], in which a priming agent that sensitizes cells toward a pro-apoptotic phenotype is administered prior to or alongside a reactivating agent so that cells replicating HIV will be induced to die. Specificity for HIV-infected cells, and HIV-induced cell death mechanisms, will be critical for this approach to be effective and clinically applicable, in order to avoid potential toxicities related to inducing apoptosis of healthy cells.

## Conclusions

It is likely that HIV kills infected CD4 T cells through a number of mechanisms, including the three described. As current and future research efforts focus on HIV eradication, insights into how HIV kills some, yet not all, infected CD4 T cells, thereby allowing latency to be established, may yield novel drug-able targets against which either molecular or cellular therapies might be developed. Future research is needed to elucidate the molecular mechanisms involved in protecting latently infected cells from HIV-mediated killing, even after reactivation of viral replication.

## Abbreviations

AIDS: Acquired immunodeficiency syndrome
ART: Antiretroviral therapy
GFP: Green fluorescent protein
HIV: Human immunodeficiency virus
HLAC: Human lymphoid aggregate culture
INSTI: Integrase strand transfer inhibitor
LTR: Long terminal repeat
MOMP: Mitochondrial outer membrane permeabilization
NNRTI: Non-nucleoside reverse transcriptase inhibitor
NRTI: Nucleoside reverse transcriptase inhibitor
PI: Protease inhibitor
TNF: Tumor necrosis factor
TRAIL: TNF-related apoptosis inducing ligand
TUNEL: Terminal deoxynucleotidyl transferase dUTP nick end labeling.
